# Case Report: Sequential FcRn blockade and B-cell depletion for treatment-refractory relapsing autoimmune encephalitis: a three-patient case series

**DOI:** 10.3389/fimmu.2026.1687948

**Published:** 2026-04-23

**Authors:** Yao Tan, Qinqin Liu, Jie Lu, Donglin Zhu, Ming Sun

**Affiliations:** 1 Department of Emergency Medicine, The Affiliated Brain Hospital of Nanjing Medical University, Nanjing, Jiangsu, China; 2 Department of Rehabilitation, The Affiliated Suqian Hospital of Xuzhou Medical University, Suqian, Jiangsu, China; 3 Department of Neurology, The Affiliated Brain Hospital of Nanjing Medical University, Nanjing, Jiangsu, China; 4 Department of Emergency Medicine, The Affiliated Suqian Hospital of Xuzhou Medical University, Suqian, Jiangsu, China; 5 Suqian Medical Research Institute of Nanjing University Medical School, Suqian, Jiangsu, China; 6 Department of Emergency Medicine, Suqian Clinical Medical College of Yangzhou University, Suqian, Jiangsu, China; 7 Department of Emergency Medicine, Nanjing Drum Tower Hospital Group Suqian Hospital, Suqian, Jiangsu, China

**Keywords:** autoimmune encephalitis, case report, cyclophosphamide, efgartigimod, relapse/refractory, rituximab

## Abstract

**Introduction:**

Refractory autoimmune encephalitis (AE), particularly in patients experiencing recurrent relapses, presents significant therapeutic challenges and underscores the urgent need for innovative treatment strategies. Emerging evidence shows efgartigimod (EFG) rapidly clears pathogenic antibodies (days), while rituximab (RTX) suppresses new antibody production (weeks-months). Their synergistic temporal effects provide a rationale for exploring sequential EFG-RTX as a treatment strategy. Here, we describe the preliminary clinical outcomes observed in three patients with refractory AE who received this sequential regimen.

**Case presentations:**

The cohort included three refractory cases: A 30-year-old female patient diagnosed with anti-glutamate decarboxylase 65 encephalitis, who had experienced three disease relapses and presented with refractory epilepsy, a 54-year-old female with relapsed anti-leucine-rich glioma-inactivated protein 1 encephalitis showing progressive cognitive decline and psychiatric symptoms, and a 19-year-old female with relapsed anti-N-methyl-D-aspartate receptor encephalitis, characterized by severe cognitive dysfunction, psychiatric disturbances, depressed consciousness, and central hypoventilation. All patients had demonstrated resistance to both first-line immunotherapies and second-line agents including cyclophosphamide. Notably, administration of EFG yielded substantial clinical improvement in all cases, with sustained remission achieved following subsequent RTX and no evidence of disease relapse.

**Conclusion:**

In this small case series, sequential EFG-RTX therapy was associated with substantial clinical improvement and sustained remission in three patients with highly refractory, relapsing AE. These preliminary observations suggest that this synergistic approach may represent a viable therapeutic option for similar difficult-to-treat cases and warrants further investigation in larger, controlled studies.

## Introduction

Autoimmune encephalitis (AE) is an increasingly recognized inflammatory disorder of the central nervous system (CNS) mediated by pathogenic autoantibodies. The most common clinical subtypes include anti-N-methyl-D-aspartate receptor (NMDAR) and anti-leucine-rich glioma-inactivated 1 (LGI1) encephalitis, while relatively rare forms such as anti-glutamic acid decarboxylase 65 (GAD65) encephalitis demonstrate more heterogeneous manifestations (1). These autoantibodies target distinct neuronal antigens; those against surface or synaptic proteins (e.g., NMDAR, LGI1) directly impair synaptic function. In contrast, antibodies against intracellular antigens (e.g., GAD65) are robust diagnostic markers, though their pathogenesis may involve distinct, T-cell-driven mechanisms (1). Disorders associated with intracellular antibodies generally show a poorer response to immunotherapy than those mediated by surface antibodies (2). Notwithstanding these differences, the humoral immune response—characterized by specific antibodies and B cells—remains a central therapeutic target across AE subtypes (3, 4).

Although most patients with AE, particularly those with surface antibody-mediated forms, respond well to current first-line immunotherapies, such as corticosteroids, intravenous immunoglobulin (IVIg), and therapeutic plasma exchange (TPE), a significant proportion (approximately 20–50%) demonstrate inadequate responses (5). Escalation to second-line agents like intravenous cyclophosphamide (CTX) may be required, yet a subset of patients develop refractory AE (6). Furthermore, some experience relapses (7, 8), making these cases a considerable therapeutic challenge (6, 8).

The anti-CD20 monoclonal antibody rituximab (RTX) is a promising second-line option for refractory AE, promoting functional recovery (9) by depleting CD20^+^ B cells and inhibiting new antibody production for months (10). However, its clinical effects are delayed, typically appearing around 2 months after initiation (11). Efgartigimod (EFG) offers a rapid alternative via neonatal Fc receptor (FcRn) ​blockade, accelerating IgG degradation and often yielding symptom relief within days (12), though its transient effect necessitates repeated dosing (13–15). The complementary profiles of FcRn blockade and B-cell depletion provide a strong rationale for sequential therapy, yet real-world data in highly refractory, relapsing AE remain scarce.

Here, we present the treatment outcomes of three cases of treatment-refractory relapsing AE following sequential EFG-RTX therapy: one with anti-GAD65, one with anti-LGI1, and one with anti-NMDAR encephalitis.

## Case presentations

### Patient 1

**Figure 1:**
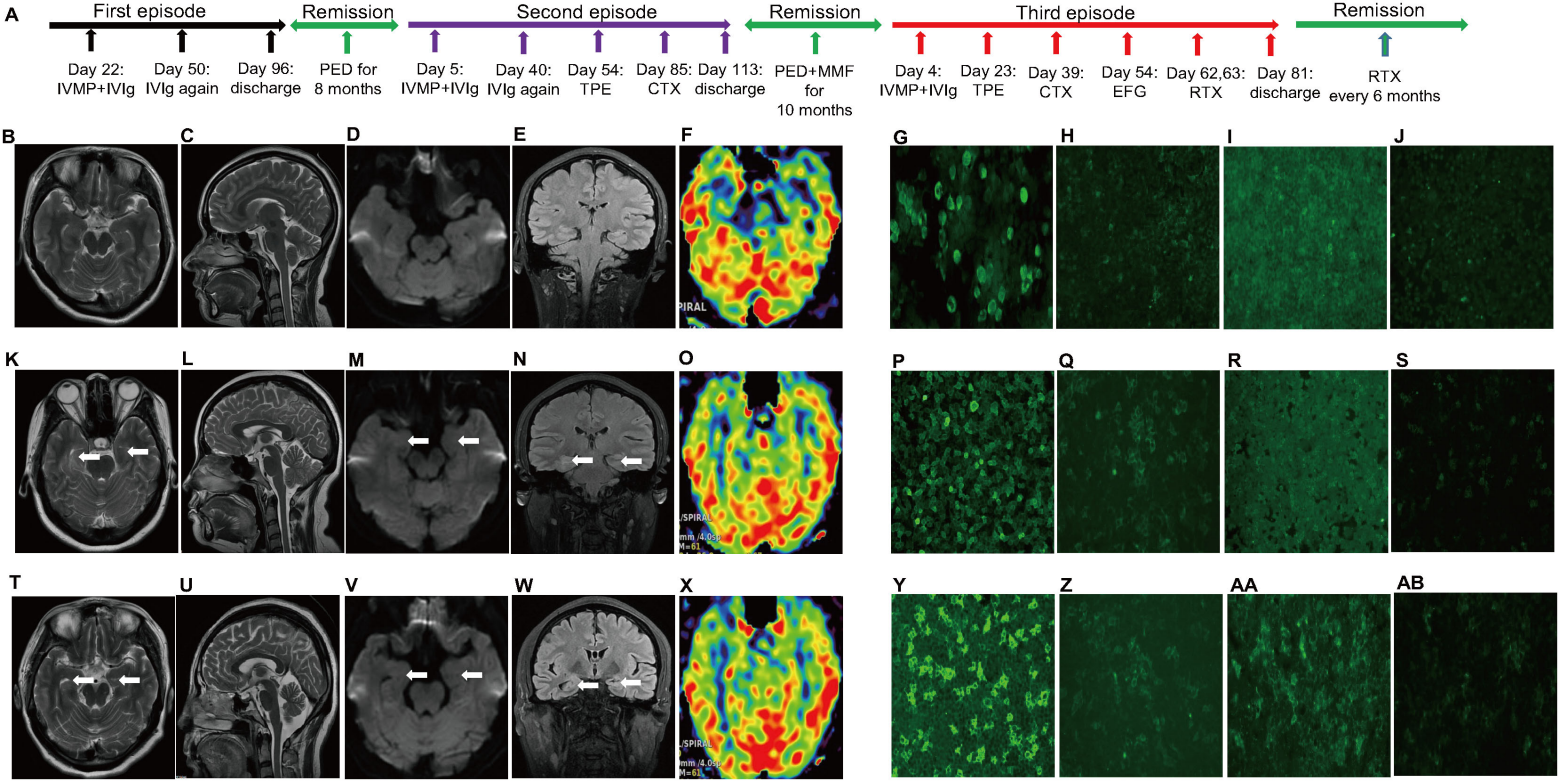
Clinical course, neuroimaging evolution, and antibody dynamics in a patient with relapsing anti-GAD65 encephalitis. **(A)**​ Timeline depicting three disease episodes, treatments, and remission periods. Relapses and remissions are marked by red and green arrows, respectively. The serial MRIs illustrate the disease’s progressive impact: unremarkable initially **(B-F)**, followed by hippocampal swelling during the second relapse [**(K-O)**, arrowheads], culminating in hippocampal atrophy in the refractory third episode **(T-X)**.​ Corresponding antibody titers quantitatively reflect disease activity: they decreased with initial therapies in Episodes 1 (serum/CSF: 1:100/1:10 to 1:10/1:3.2; G-J) and 2 (1:100/1:10 to 1:10/1:1; P-S). The catastrophic third relapse was marked by a pre-treatment titer surge (1:320/1:10; Y-Z), which declined after salvage EFG-RTX therapy [1:32/1:1; **(AA, AB)**], correlating with clinical recovery. CSF, cerebrospinal fluid; CTX, cyclophosphamide; EFG, efgartigimod; GAD65, anti-glutamic acid decarboxylase 65; IVIg, intravenous immunoglobulin; IVMP, intravenous methylprednisolone; MMF, mycophenolate mofetil; MRI, Magnetic resonance imaging; PED, prednisone; RTX, rituximab; TPE, therapeutic plasma exchange.

The first case involves a 30-year-old female patient experiencing her third episode of anti-GAD65 encephalitis. Her initial onset occurred at age 28 with sudden development of frequent epileptic seizures (10–20 episodes daily) characterized by transient consciousness impairment and limb convulsions (**Figure 1A**). Neurological examination revealed concurrent hallucinations, cognitive decline, and language comprehension difficulties (modified Rankin Scale [mRS] 4, Clinical Assessment Scale in Autoimmune Encephalitis [CASE] 9). Video-electroencephalogram (EEG) demonstrated temporal lobe-originated spike-and-wave complexes (**Supplementary Figure 1A**), while brain magnetic resonance imaging (MRI) showed no specific abnormalities (**Figures 1B–F**). The patient rapidly progressed to status epilepticus, requiring combination therapy with intravenous diazepam, carbamazepine, and valproate sodium. Routine blood tests including thyroid antibodies, rheumatologic markers, and infectious disease screening were unremarkable. Lumbar puncture revealed normal pressure and cerebrospinal fluid (CSF) parameters except for elevated immunoglobulin G (IgG). Serum and CSF anti-GAD65 antibodies were detected at 1:100 and 1:10 respectively (**Figures 1G, H**). Immunotherapy was initiated on day 22 with intravenous methylprednisolone (IVMP, 1g × 3 days) combined with IVIg (25g/day × 5 days), followed by oral prednisone (1mg/kg/day with 5mg biweekly tapering). Seizures completely resolved by day 50, though cognitive and language impairments persisted (mRS 4, CASE 6). A second IVIg course was administered due to unavailable TPE equipment. By day 81, a significant clinical improvement was observed (mRS 1, CASE 3), as evidenced by decreased antibody titers (serum 1:10, CSF 1:3.2, **Figures 1I, J**) and the presence of slow-wave bursts on EEG (**Supplementary Figure 1B**). Following 3 months hospitalization, she maintained remission for 8 months on prednisone and antiepileptic drugs (AEDs: carbamazepine + valproate), experiencing only biannual focal seizures until AED optimization (carbamazepine + valproate + lamotrigine) achieved complete seizure control. At age 29, she suffered a second episode presenting as status epilepticus (mRS 4, CASE 10) with bilateral hippocampus swelling on MRI (**Figures 1K–O**) and recurrent antibody elevation (serum 1:100, CSF 1:10, **Figures 1P, Q**). Video-EEG once again revealed spike-and-wave complexes of temporal lobe origin (**Supplementary Figure 1C**). Initial IVMP combined with IVIg therapy initiated on day 5 demonstrated a partial response, characterized by seizure control but persistent neurological symptoms (mRS 4, CASE 8). Given the incomplete response, rescue therapies were subsequently administered, including a second course of IVIg on day 40, TPE from day 54 to 61, and intravenous CTX at a dose of 750 mg/m² on day 85. These interventions resulted in moderate clinical improvement (mRS 2, CASE 4), a reduction in antibody titers (serum 1:10, CSF 1:1, **Figures 1R, S**), and the appearance of slow-wave bursts on EEG (**Supplementary Figure 1D**). Maintenance with prednisone (6 months) and mycophenolate mofetil (1500mg/day) stabilized her until 10 months post-discharge (age 30), when third relapse occurred with catastrophic presentation (mRS 4, CASE 14) and dramatic antibody surge (serum 1:320, CSF 1:10, **Figures 1Y, Z**). Cranial MRI revealed asymmetric atrophy of the bilateral hippocampus, with the right hippocampus showing medial temporal atrophy (MTA) grade 2 and the left hippocampus showing MTA grade 1 (**Figures 1T–X**). Video-EEG continues to exhibit spike complexes of temporal lobe origin (**Supplementary Figure 1E**). Sequential immunotherapy (IVMP + IVIg day 4-8, TPE day 23-27, CTX 750mg/m² day 39) showed limited efficacy until EFG (600mg day 54) induced dramatic improvement (mRS 1, CASE 3 by day 60). Consolidation with RTX (100mg + 500mg days 62-63) led to near-complete recovery by discharge (day 81: mRS 0, CASE 1) with reduced antibody titers (serum 1:32, CSF 1:1, **Figures 1AA, AB**) and a normal EEG (**Supplementary Figure 1F**). She remained relapse-free for 18 months on biannual RTX maintenance (500mg) and a simplified AED regimen. Her cognitive function was assessed with the Mini-Mental State Examination (MMSE) at the 18-month follow-up, yielding a score of 22, indicative of mild cognitive impairment.

### Patient 2

**Figure 2:**
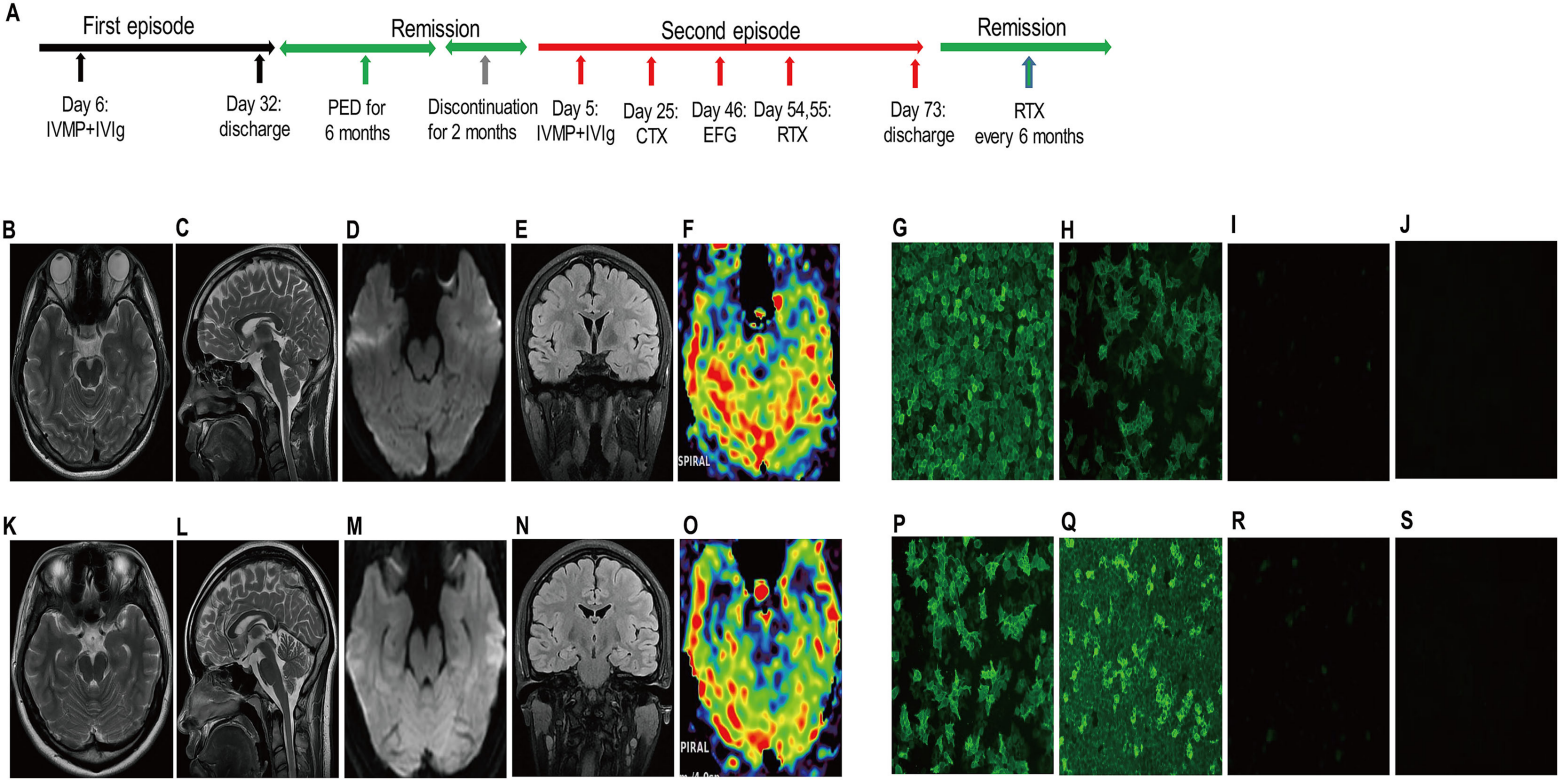
Clinical timeline, neuroimaging features and antibody profiles in a patient with relapsing anti-LGI1 encephalitis. **(A)**​ Clinical timeline of two disease episodes. Notably, MRI remained structurally normal in both episodes **(B-F, K-O)** despite severe clinical symptoms, a characteristic feature.​ In contrast, serum and CSF anti-LGI1 antibody titers served as sensitive biomarkers of disease activity. They were elevated at relapse [Episode 1: 1:320/1:32, **(G-H)**; Episode 2: 1:100/1:100, **(P-Q)**] and became negative or near-negative [1:1/negative, **(I, J, R, S)**] following immunotherapy, with the second episode’s serological response corresponding to the administration of sequential EFG-RTX. CSF, cerebrospinal fluid; CTX, cyclophosphamide; EFG, efgartigimod; IVIg, intravenous immunoglobulin; IVMP, intravenous methylprednisolone; LGI1, leucine-rich glioma-inactivated protein 1; PED, prednisone; RTX, rituximab.

The second case involves a 54-year-old previously healthy male who developed rapidly progressive dementia and behavioral abnormalities over 1 month prior to admission at age 53, resulting in complete functional dependence (mRS 4, CASE 12, **Figure 2A**). Initial evaluation showed unremarkable thyroid function, rheumatologic markers, and infectious disease screening. Brain MRI revealed no structural abnormalities (**Figures 2B–F**), while 24-hour video-EEG demonstrated slow-wave predominance across all leads (**Supplementary Figure 2A**). CSF analysis was normal except for positive anti-LGI1 antibodies (serum 1:320, CSF 1:32, **Figures 2G, H**). Immunotherapy was initiated on hospital day 6 with IVMP 1g for 3 days combined with IVIg 30g/day for 5 days, followed by oral prednisone starting at 60mg with biweekly 5mg tapering, achieving significant clinical improvement by week 4 (mRS 1, CASE 3). After completing 6 months of oral prednisone maintenance therapy, the patient’s follow-up antibody tests were all negative (**Figures 2I, J**), the follow-up video-EEG showed near-normal results (**Supplementary Figure 2B**). However, symptom relapse occurred 2 months after discontinuation of oral prednisone, featuring recurrent dementia, psychiatric symptoms, somnolence, and characteristic faciobrachial dystonic seizures (FBDS; mRS 4, CASE 14). Repeat evaluation demonstrated similar video-EEG abnormalities (**Supplementary Figure 2C**), brain MRI revealed no structural abnormalities (**Figures 2K–O**), and anti-LGI1 antibody titers were elevated (serum 1:100, CSF 1:100, **Figures 2P, Q**). The combination with IVMP + IVIg (initiated on day 5) only resolved FBDS by week 2 (mRS 4, CASE 11), while severe hyponatremia (115mmol/L), aggressive behavior, and labile blood pressure contraindicated TPE. CTX (750mg/m² starting day 25) provided minimal benefit (mRS 4, CASE 11 at week 4), prompting EFG administration (10mg/kg on day 46) which resulted in rapid clinical improvement (seizure cessation by day 49, mRS 2, CASE 4; further improvement to mRS 1, CASE 2 by day 53). Consolidation therapy with RTX (100mg + 500mg on days 54-55) led to complete remission by discharge on day 73 (mRS 0, CASE 1). Subsequent maintenance with prednisone taper and biannual RTX (500mg) sustained a disease-free status at the 16-month follow-up, evidenced by negative antibody levels (**Figures 2R, S**), a normalized EEG (**Supplementary Figure 2C**), and a cognitive assessment score of 27 on the MMSE, indicating substantial recovery with possible subtle residual deficits.

### Patient 3

**Figure 3:**
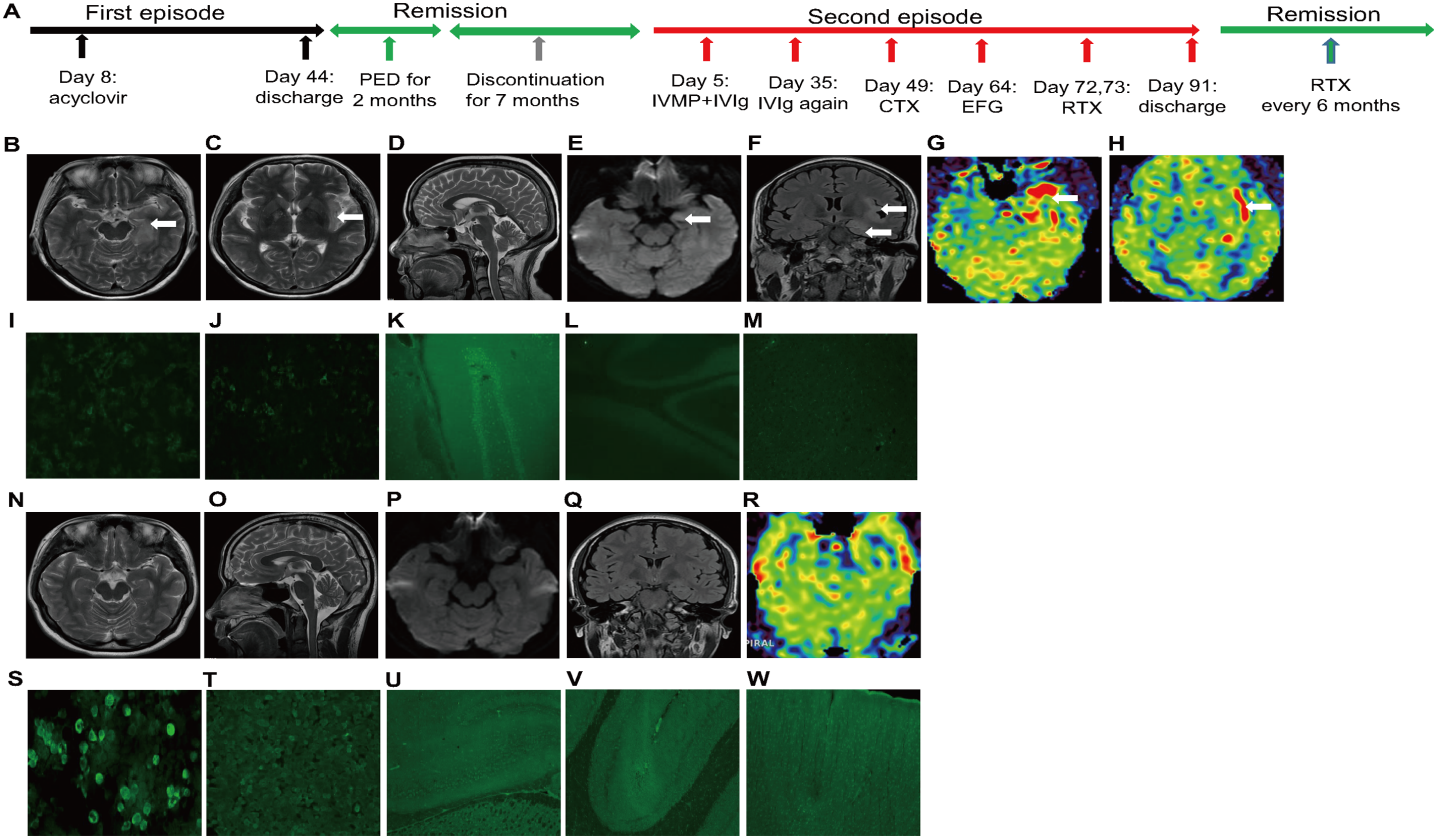
Clinical timeline, neuroimaging features and antibody profiles in a patient with relapsing anti-NMDAR encephalitis. **(A)**​ Disease course. The first episode presented with left temporo-insular lesions and hyperperfusion [**(B-H)**, arrow] alongside low-level antibodies [serum/CSF: 1:10/1:3.2; **(I, J)**] and a positive tissue-based assay **(K-M)**. The second, severe relapse demonstrates a key clinical-radiological dissociation: MRI was normal **(N-R)** despite catastrophic symptoms and significantly elevated antibody titers [1:100/1:32; **(S, T)**, with positive TBA **(U-W)**].​ The clinical response to EFG-RTX occurred in this context of seropositive, MRI-negative disease activity. CSF, cerebrospinal fluid; CTX, cyclophosphamide; EFG, efgartigimod; IVIg, intravenous immunoglobulin; IVMP, intravenous methylprednisolone; NMDAR, N-methyl-D-aspartate-receptor; PED, prednisone; RTX, rituximab.

The third case involves a 19-year-old female with no prior chronic medical history. At age 18, she developed amnesia and confusion following a febrile upper respiratory infection (**Figure 3A**). Initial evaluation revealed normal hematological parameters, though CSF analysis showed elevated leukocyte count (110/ul) with normal protein and electrolytes. Cranial MR imaging revealed multiple abnormal signal intensities in the left temporal insular lobe, and arterial spin labeling demonstrated corresponding hyperperfusion in the same region (**Figures 3B–H**). The initial video-EEG revealed the presence of fast waves (**Supplementary Figure 3A**). Serum and CSF antibody screenings, including anti-NMDAR antibodies, were negative. However, next-generation sequencing of the CSF pathogen profile indicated herpes simplex virus type 1 infection (detected sequence reads: 161). She received a 5-week course of acyclovir due to slow symptom resolution, after which CSF leukocytes normalized. Repeated screening for AE demonstrated that the serum anti-NMDAR antibody titer was 1:10 and the CSF anti-NMDAR antibody titer was 1:3.2 (**Figures 3I, J**). However, tissue-based assays (TBA) performed on rat cerebellum, hippocampus, and cerebral cortex tissues showed scattered fluorescence signals (**Figures 3K–M**). Moreover, Re-examination of the video-EEG revealed the presence of theta wave activity (**Supplementary Figure 3B**). Therefore, she was diagnosed with secondary AE following a herpesvirus infection. Given the absence of significant symptoms at the time of diagnosis, she was initiated on oral prednisone therapy at a daily dose of 60 mg. However, 2 months later, she discontinued the treatment on his own due to the development of acne. Seven months later (age 18), she presented with acute-onset confusion, profound cognitive impairment, and decreased consciousness (mRS 4, CASE 14) within 1 week of another upper respiratory infection. Admission tests (thyroid antibodies, rheumatologic markers, infectious screening) were unremarkable. Brain MRI revealed no evidence of structural abnormalities (**Figures 3N–R**). Repeat lumbar puncture showed no CSF abnormalities. By day 3, she required mechanical ventilation for refractory hypoxemia and vasopressor support (dopamine + metaraminol) for hypotension, necessitating nasogastric feeding. Repeated video-EEG detected the presence of widespread slow wave activity (**Supplementary Figure 3C**). On day 5, autoimmune antibody testing revealed significantly elevated antibodies: serum anti-NMDAR 1:100 and CSF anti-NMDAR 1:32 (**Figures 3S, T**), with positive TBA confirmation (**Figures 3U–W**). Immunotherapies were immediately started with high-dose IVMP (1g/day × 3 days) and IVIg (25g/day × 5 days), followed by a prednisone taper (halved every 5 days). Symptoms gradually improved; by day 26, ventilator weaning was attempted (mRS 3, CASE 6). However, transitioning to oral prednisone (60mg) on day 30 triggered symptom recurrence, requiring resumption of ventilatory and vasopressor support (mRS 4, CASE 14). A second IVIg cycle (25g/day × 5 days) was administered on day 35. By day 49, with no clinical improvement (mRS 4, CASE 14) and unstable cardiorespiratory status precluding TPE, intravenous CTX was initiated. However, lack of response persisted after the first CTX dose. On day 64, intravenous EFG (600mg) was administered, resulting in rapid clinical improvement: ventilator and vasopressor independence by day 68 (mRS 3, CASE 5), resolution of psychiatric symptoms and independent ambulation by day 72 (mRS 2, CASE 2), though mild memory deficits remained. Two-dose RTX therapy (100mg on day 72, 500mg on day 73) was initiated. She achieved a full functional recovery (mRS 0, CASE 2) with a normal EEG (**Supplementary Figure 3D**) at the time of discharge on day 91. Prednisone was tapered by 5mg every two weeks until discontinuation, supplemented with semi-annual RTX infusions (500mg/dose). Disease-free status was maintained at the 13-month follow-up, as indicated by sustained remission and an MMSE score of 29, reflecting excellent cognitive recovery.

## Discussion

This case series provides early clinical experience with sequential EFG-RTX therapy in a challenging cohort of patients with treatment-refractory relapsing AE. Our observations support the practical application of the mechanistically rational ‘fast-acting + sustained-effect’ strategy in a real-world setting where conventional therapies have failed. All three patients, refractory to standard therapies, showed a consistent pattern: rapid symptomatic improvement after a single dose of EFG, with subsequent RTX administration (given at 6-month intervals) suggesting a synergistic effect ​in sustaining remission and preventing further relapse.

Clinically, anti-GAD65 encephalitis often presents with temporal lobe epilepsy (particularly in refractory cases), cognitive impairment, and behavioral changes (16), with epileptic seizures frequently being the initial and predominant symptom (17), as seen in Patient 1. High anti-GAD65 antibody titers typically correlate with syndromes like chronic epilepsy and limbic encephalitis (18), matching this patient’s presentation. While first-line immunotherapy shows some efficacy for non-epileptic symptoms, its effect on seizures is limited, with approximately 80% of patients continuing to experience seizures—a rate higher than in other AE subtypes (19, 20). Seizures in anti-GAD65 encephalitis are typically refractory to conventional AEDs (21), whereas immunotherapy may offer a more effective approach for seizure control (18). Consistent with this, the patient’s seizures terminated rapidly after EFG administration. This observation, along with a recent study (15), suggests that​ anti-GAD65 encephalitis may​ respond notably to EFG. Furthermore, the sustained remission observed in this and other reports (22, 23) is consistent with the hypothesis that ​RTX contributes to long-term remission by suppressing *de novo* antibody production.

Anti-LGI1 encephalitis presents with FBDS, cognitive impairment, psychiatric symptoms, and hyponatremia. Its pathogenesis is driven by intrathecal B-cell immunity, evidenced by clonal expansion and somatic hypermutation of CSF B-cells (24). Persistent intrathecal IgG synthesis suggests compartmentalized B-cell activation (24), which may perpetuate disease and underlie relapses. Although most patients respond initially to immunotherapy (25, 26), 15–50% relapse (median 5 months) (8, 27–31), particularly those with delayed treatment (8) or persistent CSF antibodies (28). Relapses are associated with poorer outcomes, including refractory cognitive deficits (31), and may be exacerbated by high antibody titers (8). Conventional maintenance therapies show limited efficacy in refractory cases (29). The temporal synergy of FcRn blockade and B-cell depletion represents an emerging strategy: EFG can rapidly reduce pathogenic antibodies within days (32, 33), while RTX—despite limited blood-brain barrier (BBB) penetration (24)—may modulate intrathecal immunity, as suggested by early EEG improvements (34).

Anti-NMDAR encephalitis, the most prevalent AE, manifests with cognitive impairment, seizures, psychiatric symptoms, and sometimes life-threatening central hypoventilation. Despite increased clinical experience, mortality and relapse rates remain concerning, with a subset progressing to refractory disease (35). Both EFG and RTX have shown therapeutic potential. Multicenter studies indicate EFG may be superior to IVIg in reducing antibody titers (36), with rapid clinical improvement reported in refractory cases (13, 14). RTX may not only mitigate acute symptoms but also provide long-term benefit through sustained B-cell depletion, potentially reducing relapse risk when initiated early (9, 37).

Notably, while existing studies emphasize the temporal dichotomy between EFG’s rapid action and RTX’s sustained effects, emerging evidence (38)—including our observations in a pathophysiologically diverse cohort ​(encompassing surface antibody-mediated anti-NMDAR/LGI1 and intracellular antigen-associated anti-GAD65 encephalitis)—invites a more nuanced model. First, data confirm RTX can exert immunomodulatory effects within 1–2 weeks (34, 39–42), challenging the view of an exclusively delayed action. Second, in our cases, significant symptom improvement occurred within 1 month after a single dose of EFG, suggesting synergistic early efficacy where EFG’s immediate IgG clearance may potentiate RTX’s emerging B-cell modulation. Critically, this consistent early response across mechanistically distinct AE subtypes supports a unified therapeutic rationale centered on peripheral humoral immunomodulation. In surface antibody-mediated AE, the benefit derives directly from pathogenic antibody clearance and B-cell depletion. In intracellular antibody-associated AE (e.g., anti-GAD65), where T-cell driven pathology is implicated, the benefit may arise indirectly through the attenuation of the antibody-mediated humoral response that supports neuroinflammation and T-cell activation (43).​ This real-world evidence supports a revised kinetic profile wherein both agents contribute to the early response. The impact of this peripheral immunomodulatory strategy ​on CNS-predominant disease, despite theoretical BBB limitations, may be explained by: (1) BBB disruption in active disease; (2) the dual peripheral action preventing replenishment of CNS-targeting immune mediators; and (3) a “suction effect” promoting efflux of inflammatory mediators from the CNS, ultimately facilitating immune homeostasis.

The cognitive trajectory in these patients warrants specific attention alongside overall clinical improvement. While sequential therapy resolved major neurological and psychiatric symptoms, persistent memory deficits were observed—consistent with existing evidence indicating that cognitive recovery in AE is frequently incomplete and may occur independently of serum antibody normalization (44, 45). This persistence may be attributed to mechanisms not fully addressed by peripheral antibody reduction. Emerging evidence highlights CNS-compartmentalized immunity as a key factor, where compartmentalized, tissue-resident memory T cells can sustain local inflammation (46), and long-lived plasma cells within CNS niches may continue local antibody production (47). Notably, cognitive impairments in AE have been directly linked to intrathecal humoral immune activity including autoimmune antibodies and oligoclonal bands (48), providing a plausible mechanistic basis for both the residual cognitive dysfunction observed and the underlying relapse risk. It is also crucial to acknowledge that the MMSE, used here, is a global screening tool insensitive to the specific fronto-subcortical and hippocampal deficits common in AE (49, 50). Future prospective studies should therefore incorporate comprehensive neuropsychological batteries (51, 52) and longitudinal CSF immune profiling to directly assess treatment impact on the intrathecal compartment.

All these patients described the combined regimen as providing rapid symptom control and durable remission after conventional therapies failed, expressing profound gratitude for this transformative intervention.

The findings of this case series should be interpreted within the context of its inherent limitations. These include the small sample size (n=3) and heterogeneity of AE subtypes, which limit generalizability. The observational design and lack of a control group preclude definitive causal attributions, and the potential confounding effects of delayed responses to extensive prior immunotherapies cannot be excluded. Furthermore, cognitive assessment relied primarily on the MMSE, and the absence of longitudinal CSF immune profiling limits our ability to directly assess the therapy’s impact on the intrathecal compartment.

Despite these limitations, this case series provides preliminary clinical experience suggesting the potential utility of sequential EFG-RTX therapy for relapsed or refractory AE. The observed trajectory aligns with the complementary mechanisms of FcRn blockade and B-cell depletion. This approach warrants further investigation in larger, prospective studies to validate efficacy and define optimal protocols.

## Data Availability

The original contributions presented in the study are included in the article/**Supplementary Material**. Further inquiries can be directed to the corresponding authors.
