# Supplementary material for: Case Report: Sequential FcRn blockade and B-cell depletion for treatment-refractory relapsing autoimmune encephalitis: a three-patient case series
Source: Front Immunol. 2026 Apr 23;17:1687948. doi: 10.3389/fimmu.2026.1687948 (PMC13149427; doi:10.3389/fimmu.2026.1687948)
Supplement: Supplementary file 4 [file SupplementaryFile1.pdf]

Figure S1. Video-electroencephalography (EEG) changes in Patient 1 before and after immunotherapy across three anti-GAD65 encephalitis episodes. The first episode manifested with tonic-clonic seizures, correlating with bilateral asynchronous temporal high-amplitude 3.0–3.5 Hz spike-and-wave complexes on EEG (**A**); post-immunotherapy, seizures resolved with residual 1.5–2.5 Hz slow-wave bursts (**B**). The second episode exhibited identical pre- and post-treatment EEG patterns to the first episode(**C** and **D**). During the third episode, EEG again demonstrated bilateral temporal-origin spike-and-wave discharges (**E**), which normalized completely following immunotherapy (**F**).

Figure S2. Video-electroencephalography (EEG) changes in Patient 2 before and after immunotherapy across two anti-LGI1 encephalitis episodes. During the first episode, the patient exhibited no seizures, with EEG demonstrating generalized 6–7 Hz medium-amplitude slow-wave predominance across all leads (**A**); post-immunotherapy, EEG returned to near-normal patterns (**B**). The second episode showed identical pre- and post-treatment EEG characteristics to the first episode(**C** and **D**).

Figure S3. Video-electroencephalography (EEG) changes in Patient 3 before and after immunotherapy across two anti-NMDAR encephalitis episodes. During the first episode, the patient presented without seizures(**A**); EEG showed generalized mild excess of 15–20 Hz low-amplitude fast waves, which transitioned to bilateral anterior (prefrontal, frontal, central, parietal, midline) 5–7 Hz theta rhythm bursts (short-to-long

duration) post-immunotherapy (**B**). In the second episode, pre-treatment EEG revealed moderate-to-severe abnormalities with frequent generalized medium-to-high amplitude 2.5–3.5 Hz slow waves (predominantly in bilateral frontal and temporal regions, with Fz-A1 lead artifact, **C**), while post-treatment EEG returned to normal (**D**).
